# Iron and iron/manganese ratio in forage from Icelandic sheep farms: relation to scrapie

**DOI:** 10.1186/1751-0147-48-16

**Published:** 2006-08-31

**Authors:** KB Gudmundsdóttir, S Sigurdarson, J Kristinsson, T Eiríksson, T Jóhannesson

**Affiliations:** 1 Chief Veterinary Office, Section for Animal Diseases. Institute for Experimental Pathology, University of Iceland, Keldur, 112 Reykjavík, Iceland; 2 Institute of Pharmacy, Pharmacology & Toxicology, Department of Pharmacology and Toxicology, University of Iceland, Hofsvallagata 53, 107 Reykjavík, Iceland; 3 Department of Animal and Land Resources, Agricultural University of Iceland, Keldnaholt, 112 Reykjavík, Iceland

## Abstract

This study was undertaken in order to examine whether any connection existed between the amounts of iron in forage and the sporadic occurrence of scrapie observed in certain parts of Iceland. As iron and manganese are considered antagonistic in plants, calculation of the Fe/Mn ratios was also included by using results from Mn determination earlier performed in the same samples. Forage samples (n = 170) from the summer harvests of 2001–2003, were collected from 47 farms for iron and manganese analysis. The farms were divided into four categories: 1. *Scrapie-free farms in scrapie-free areas *(n = 9); 2. *Scrapie-free farms in scrapie-afflicted areas *(n = 17); 3. *Scrapie-prone farms *(earlier scrapie-afflicted, restocked farms) (n = 12); 4. *Scrapie-afflicted farms *(n = 9). Farms in categories 1 and 2 are collectively referred to as *scrapie-free farms*. The mean iron concentration in forage samples from scrapie-afflicted farms was significantly higher than in forage samples from farms in the other scrapie categories (P = 0.001). The mean Fe/Mn ratio in forage from scrapie-afflicted farms was significantly higher than in forage from scrapie-free and scrapie-prone farms (P < 0.001). The results indicated relative dominance of iron over manganese in forage from scrapie-afflicted farms as compared to farms in the other categories. Thus thorough knowledge of iron, along with manganese, in soil and vegetation on sheep farms could be a pivot in studies on sporadic scrapie.

## Background

The prion protein (PrP) occurs naturally in most organs. It is believed to have a role in copper metabolism and possibly also in oxidative defense and functions of the central nervous system. The protein is present in both a free and a glycosylated form, bound to cell membranes. In prion diseases (also called transmissible spongiform encephalopathies (TSEs)), the prion protein takes on a pathological, misfolded form (often called PrP^sc^), leading to depositions of extracellular aggregates and spongiform degeneration (vacuolation) in the brain. TSEs are always lethal. As the name implies, a distinguishing feature of TSEs is their transmissibility between individuals of the same species, or even between individuals of different species [1-4].

Scrapie in sheep and goats is one of the best known TSEs. Scrapie in sheep has for decades been one of the most costly diseases in Icelandic livestock [5]. In spite of stamping out and systemic preventive measures, the disease still occurs sporadically on sheep farms in Iceland each year, especially on farms in a few areas in southern, northern and eastern parts of the country [6]. This sporadic occurrence of scrapie may indicate an environmental factor or factors which may be predisposing to the development of clinical scrapie, by influencing the conversion of PrP to PrP^sc^, or otherwise influencing the pathogenesis of the disease [2]. Amounts of trace elements in the feed might represent such factors. *Jóhannesson et al*. [6] found that forage samples from scrapie-free farms in Iceland contained on average significantly more manganese than forage samples from scrapie-afflicted farms but no difference could be found between the copper concentration in forage samples from sheep farms of different scrapie categories (see definition below). In another study these authors found that selenium, although being generally low in Icelandic forage, did not differ in amount between forage samples from scrapie-free, scrapie-prone or scrapie-afflicted farms [7]. Nor was any significant difference found in the amounts of molybdenum (or sulphur) in forage, between farms of different scrapie categories [8].

*Adriano *[9] has described a biochemical antagonism between iron and manganese in plants and *Jóhannesson et al*. [6] have, as mentioned, found significantly higher amounts of manganese in forage of sheep on scrapie-free farms than on farms in the other categories. The primary aim of the present study was accordingly to study whether higher amounts of manganese would be reciprocated by lower amounts of iron, or alternatively lower amounts of manganese by higher amounts of iron, in the forage of sheep on farms in the three scrapie categories.

## Materials and methods

### Categories of farms and collection of forage samples

A total of 170 forage samples, from the summer harvests of 2001, 2002 and 2003, consisting mostly of round bale silage (dry matter content 30–70%), but also a few samples of hay (dry matter content ≥80%), were collected from 47 sheep farms, for analysis of iron concentration. The samples were collected from cultivated home fields, and preferably from both old and more recently cultivated plots, respectively, as previously described by *Jóhannesson et al*. [6]. About three different forage samples (two to five) were collected on each farm. In general the forage samples mainly consisted of the following grass species: Kentucky bluegrass (*Poa pratensis*), red fescue (*Festuca rubra*), tufted hairgrass (*Deschampsia caespitosa*) and bentgrass (*Agrostis *species). Timothy (*Pleum pratense*) was in a few samples, especially from more recently cultivated fields. Annual bluegrass (*Poa annua*) was not uncommon in samples from older fields.

The farms were divided into four categories according to scrapie status: 1. *Scrapie-free farms in scrapie-free areas*: Nine farms in counties or parts of counties where scrapie has never been registered. 2. *Scrapie-free farms in scrapie-afflicted areas*: Seventeen farms where scrapie has never been diagnosed or prior to 1960 and then restocked with healthy sheep, but located in counties or parts of counties afflicted by scrapie. 3. *Scrapie-prone farms*: Twelve farms afflicted by scrapie after 1980 and afterwards restocked with healthy sheep in accordance with government regulations. 4. *Scrapie-afflicted farms*: Nine farms where scrapie was diagnosed during the research period (2001–2004). Farms in categories 1 and 2 are collectively referred to as *scrapie-free farms*. The location of the farms is shown in a previous publication [6].

**Figure 1:**
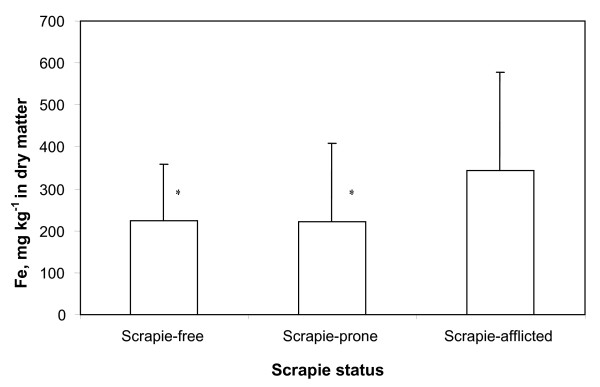
Mean concentration of iron (Fe) (mg kg^-1 ^in dry matter) in forage samples from 47 scrapie-free, srapie-prone and scrapie-afflicted farms. Vertical bars show the standard deviation. *significantly lower concentration than in samples from scrapie-afflicted farms (P = 0.001).

### Preparation of samples and analysis of iron

The preparation of the forage samples and the iron analysis was carried out as described for copper and manganese by *Jóhannesson et al*. [6]. First, the samples were dried at 65°C in a forced air oven for 48 hours. They were then stabilized at room temperature and milled in a hammer mill to pass a 1 mm screen. The milled samples were then weighed for iron analysis and to estimate dry matter content. The samples were now digested by boiling in concentrated HNO_3 _(Merck, Suprapur, Merck KgaA, Darmstadt, Germany) overnight and analysed for iron by ICP optical emission spectrometry using Spectroflame D sequential instrument (Spectra, Analytical Instruments, Kieve, Germany).

Three analytical samples were taken from each forage sample. Individual intra sample results did not differ more than ± 5%. The iron concentration (mean of measurements of three analytical samples) is expressed as mg kg^-1 ^dry matter. Leaves of poplar (certified reference material NCS CC 73350, China National Analysis Center for Iron and Steel, China, supplied from LGC Promochem, Borås, Sweden) were applied as an external standard.

### Statistical analysis

The iron concentration in the forage samples as well as the iron/manganese ratios were not normally distributed but did pass the Kolmogorov-Smirnov test for normality after transformation to logarithmic values. One way analysis (ANOVA) was used for comparison of the logarithmic values of the iron concentrations as well as the logarithmic values of the iron/manganese ratios in forage samples from farms in different categories. The Student-Newman-Keul's test was used for all pairwise comparisons.

## Results

The iron concentration in the forage samples ranged from 57 mg kg^-1 ^to 1379 mg kg^-1^. In 14 of the samples (ca. 8%) the iron concentration was less than 100 mg kg^-1 ^and in three of the samples (ca. 1.7%) it was above 1000 mg kg^-1^. It was considered that the three samples with iron concentration above 1000 mg kg^-1 ^were contaminated by soil, and these samples were thus excluded from further processing. The mean iron concentration in forage from scrapie-free farms (categories 1 and 2 combined) was 223 mg kg^-1 ^(91 samples). On scrapie-prone farms the mean iron concentration was 221 mg kg^-1 ^(40 samples), and on scrapie-afflicted farms the mean iron concentration was 343 mg kg^-1 ^(36 samples). The mean iron concentration was thus significantly higher in forage from the scrapie-afflicted farms than in forage from farms of the other scrapie categories (P = 0.001) (Fig. 1). The mean iron concentration in forage from scrapie-free farms in scrapie-free areas was 193 mg kg^-1 ^(27 samples), compared to the mean iron concentration of 236 mg kg^-1 ^in the forage from scrapie-free farms in scrapie-afflicted areas (64 samples). The difference between these two categories was however not statistically significant (P > 0.05).

**Figure 2:**
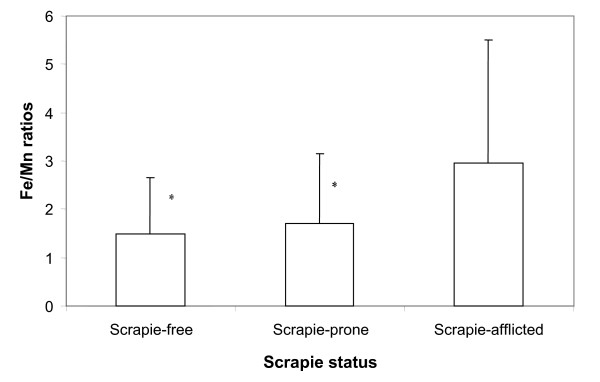
The calculated mean iron/manganese ratios in forage samples from 47 scrapie-free, scrapie-prone and scrapie-afflicted farms. Vertical bars show the standard deviation. *significantly lower ratio than in samples from scrapie-afflicted farms (P < 0.001).

By using the results from the manganese determination of the same forage samples [6], the iron/manganese (Fe/Mn) ratios were calculated for samples from farms of different scrapie categories. These calculations showed that the mean Fe/Mn ratio was 1.50 in forage from scrapie-free farms (categories 1 and 2 combined); 1.70 in forage from scrapie-prone farms and 2.68 in forage from scrapie-afflicted farms. The mean Fe/Mn ratio in forage from the scrapie-afflicted farms was thus significantly higher than in the forage from scrapie-free and scrapie-prone farms, and the difference was highly significant (P < 0.001) (Fig. 2). In forage from scrapie-free farms in scrapie-free areas the mean Fe/Mn ratio was 1.09, whereas the mean Fe/Mn ratio was 1.67 in forage from scrapie-free farms in scrapie-afflicted areas. The difference was however not statistically significant (P > 0.05).

## Discussion

The amounts of soluble iron in Icelandic soil are known to be high [10]. The iron concentration in grass (and forage made from grass) in the country is accordingly high. The high concentrations of soluble iron in soil in Iceland can probably influence the absorption of other trace elements in plants, such as manganese, copper, zinc and cobalt, which, like iron, are predominantly absorbed as divalent cations. Icelandic forage is also known to contain somewhat low amounts of zinc and cobalt, although no difference was seen between the amounts of these trace elements in forage from farms of different scrapie categories [11]. This might nevertheless be of importance for the general health status of the livestock. There is, however, at the present time no indication of manganese or copper deficiency in grass or sheep in Iceland [6,12]. This might be ascribed to the high levels of soluble manganese and copper in Icelandic soil [[10], *Th. Gudmundsson*, personal communication 2006]. In this context it is of interest that the high iron levels found in forage from scrapie-afflicted farms border on the toxic levels of the metal for plants [13].

The mean iron concentration in forage samples from scrapie-afflicted farms was significantly higher than in forage samples from farms in the other scrapie categories (Fig. 1). The forage samples from scrapie-afflicted farms were relatively few (36 in number), which is a small number for the statistical analysis. On the other hand, the mean iron concentration in forage samples from scrapie-prone farms, which were of a similar number (40 samples), was also significantly lower than in samples from scrapie-afflicted farms, and did in the same way not differ significantly from the mean iron concentration in forage from scrapie-free farms (categories 1 and 2 combined). The mean iron concentration in forage samples from scrapie-free farms in scrapie-free areas (only 27 samples) was much lower than in forage samples from scrapie-free farms in scrapie-afflicted areas (64 samples). The difference was however not significant for the number of samples included in the study. The results thus indicated that the iron concentration in the forage differed between farms of different scrapie categories, and that the concentration was highest in forage samples from farms where scrapie was present during the research period.

We have previously found that the mean manganese concentration was significantly lower in forage samples from scrapie-afflicted farms than in forage samples from scrapie-prone farms and scrapie-free farms (categories 1 and 2 combined) [6]. How this might affect occurrence of clinical scrapie is not known. In the previous publication we forwarded an idea on how high amounts of manganese specially in the gastrointestinal tract might retard or prevent the entry of prion proteins through the mucosal epithelium, and that high amounts of copper might have an opposite effect [6].

In this context, it is of interest that in plants, manganese and iron act as biochemical antagonists and may compete with each other with regard to absorption from soil and enzyme binding [9,13]. According to *Adriano *[9], the Fe/Mn ratio should be in the range 1.5 – 2.5 in healthy plants. If this ratio exceeds 2.5, it suggests a relative dominance or overactivity of iron compared to manganese, and if it is less than 1.5 it suggests a relative dominance or overactivity of manganese over iron. The Fe/Mn ratios in forage in the present study suggest that in the forage from scrapie-afflicted farms iron may be predominant over manganese, in forage from scrapie-prone and scrapie-free farms (categories 1 and 2 combined), the the two metals are in balance (Fig. 2), whereas in forage from scrapie-free farms in scrapie-free areas manganese may dominate over iron.

The results presented here would indicate that high amounts of iron in the forage of sheep might somehow premise the occurrence of clinical scrapie through lower, albeit not deficient, levels of manganese. Whether or not high amounts of iron in the forage might per se, or by other mechanisms, have any such effect awaits further research.

In conclusion, the forage samples from scrapie-afflicted farms contained significantly more iron and significantly less manganese than the forage samples from scrapie-free and scrapie-prone farms, indicating a relative dominance of iron over manganese in forage from scrapie-afflicted farms as compared to farms in the other categories. A more detailed study of iron, along with manganese, in the soil and vegetation on sheep farms might be the key to understand why scrapie is recurrently diagnosed on some farms and yet never occurs on certain neighbouring farms.
